# Near Scale-Free Dynamics in Neural Population Activity of Waking/Sleeping Rats Revealed by Multiscale Analysis

**DOI:** 10.1371/journal.pone.0012869

**Published:** 2010-09-28

**Authors:** Leonid A. Safonov, Yoshikazu Isomura, Siu Kang, Zbigniew R. Struzik, Tomoki Fukai, Hideyuki Câteau

**Affiliations:** 1 Laboratory for Neural Circuit Theory, RIKEN BSI, Wako, Japan; 2 Brain Science Institute, Tamagawa University, Machida, Japan; 3 Department of Bio-System Engineering, Graduate School of Science and Engineering, Yamagata University, Yonezawa-shi, Japan; 4 Educational Physiology Laboratory, Graduate School of Education, University of Tokyo, Tokyo, Japan; 5 CREST, JST, Kawaguchi, Japan; 6 Brain and Neural Systems Team, RIKEN Computational Science Research Program, Wako, Japan; 7 Graduate School of Life Science and Science Engineering, Kyushu Institute of Technology, Kitakyushu, Japan; Indiana University, United States of America

## Abstract

A neuron embedded in an intact brain, unlike an isolated neuron, participates in network activity at various spatial resolutions. Such multiple scale spatial dynamics is potentially reflected in multiple time scales of temporal dynamics. We identify such multiple dynamical time scales of the inter-spike interval (ISI) fluctuations of neurons of waking/sleeping rats by means of multiscale analysis. The time scale of large non-Gaussianity in the ISI fluctuations, measured with the Castaing method, ranges up to several minutes, markedly escaping the low-pass filtering characteristics of neurons. A comparison between neural activity during waking and sleeping reveals that non-Gaussianity is stronger during waking than sleeping throughout the entire range of scales observed. We find a remarkable property of near scale independence of the magnitude correlations as the primary cause of persistent non-Gaussianity. Such scale-invariance of correlations is characteristic of multiplicative cascade processes and raises the possibility of the existence of a scale independent memory preserving mechanism.

## Introduction

An isolated neuron has a volatile memory. Its membrane potential returns to the resting value once synaptic inputs stop activating/deactivating it. A neuron in a prepared in vitro brain slice, unlike a neuron in the living brain, is virtually isolated due to lack of synaptic input. After artificial activation of such a neuron, its dynamics recovers to the original state within tens of milliseconds [1]. Although an isolated neuron can summate the history of synaptic inputs, their total history is lost immediately after a spike is fired. Once neurons form a network, however, they exhibit an amazing ability to preserve activity at different time scales. Here we reveal this phenomenon with multiscale analysis of the activity of a neuron embedded in an intact brain (in vivo). Such an ability of neuronal networks, but not of isolated neurons, to retain information at different time scales, greatly enriches their computational ability. This is because now they can make use of the information across the full space-time domain, rather than spatially but at a single temporal scale.

Close investigation of the long time scales in the neural activity was pioneered in experimental studies on neuronal assemblies cultured on a multi-electrode array (MEA) [2]–[4]. There, a conventional analysis method was used to reveal power-law scaling behavior in the histograms of the sizes of the event and inter-event intervals. Observing power-law behavior, such as 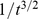, rather than an exponential decay 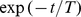, implies a lack of characteristic time scale (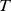) and scale-invariant behavior. Typically, scale-free characteristics are of functional significance [2]–[4]. Further studies have generalized the findings from culture preparation to slice preparation, and even to the intact brain (in vivo) of an anesthetized animal [5].

Here, we take a step further, and analyze the intact brain without anesthesia, that is the neural activity of the normally working brain. Our methodology is armed with an advanced tool to detect the presence of multiple scales in time series dynamics, such as that resulting from brain activity. In order to record the brain activity of unanesthetized animals, we developed a special chamber [6] in which rats stayed calm due to their inborn nature to favor narrow and protected places. This chamber enabled us to record neuronal activity for up to eight hours in a row. Thus, recorded data permit examination for the presence of memory in neuronal activity at time scales ranging from tens of milliseconds to at least several minutes.

We apply multiscale analysis, as it has previously been proven to be a powerful tool in unraveling the presence of multiple time scales in such diverse areas as hydrodynamic turbulence [7]–[9], human heartbeat interval fluctuations [10], [11], and stock price fluctuations [12]. By applying the method to the fluctuations of the interspike interval (ISI) of spike trains recorded from neurons in the rats' brains, we find evidence for the multiple time scales in the neural activity.

Typically for wild rats, our rats in the chamber spontaneously alternate between waking and sleeping. The observed ISI fluctuations exhibit strong non-Gaussianity, which chiefly results from the shape of the ISI distribution and its substantial autocorrelation. A close analysis reveals the following. (1) The non-Gaussianity is significantly larger during waking than sleeping. (2) The non-Gaussianity characteristics are found not only in a single neuron but also in a population of neighboring neurons, suggesting that the long time scales reside in the network. (3) Scale invariant, multiplicative cascade-like properties are observed in an average sense, accompanied by scale-invariant two-point correlations and suggesting preservation of a memory-preserving mechanism across a large range of temporal resolutions.

## Results

### Multiscale fluctuation analysis

**Figure 1:**
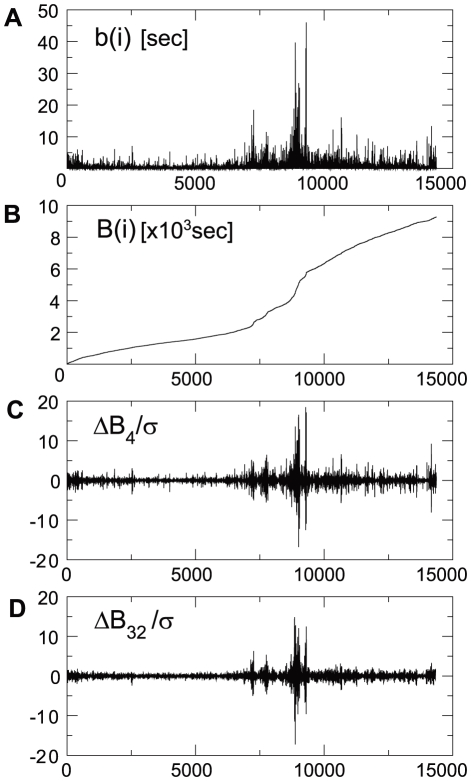
Illustration of the method to extract the non-Gaussianity in the ISI fluctuations. (A) ISI's of a spike train of a neuron are displayed chronologically. (B) Cumulated ISI's. (C)(D) ISI fluctuations were calculated by detrending (defined in the text) at 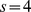 (C) and 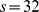 (D). The spike train was taken from a cortical neuron #12 when the animal was awake.

**Figure 2:**
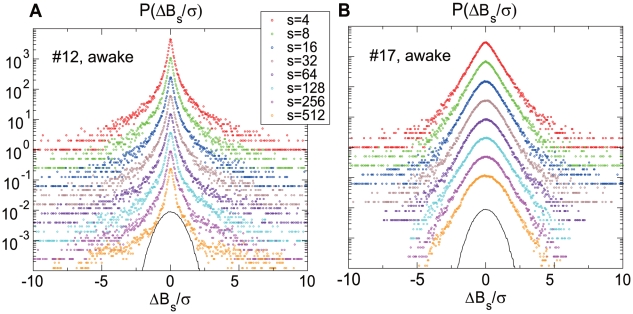
The probability distribution function (PDF) of the normalized ISI fluctuations. PDFs calculated at eight different coarse graining scales are displayed for neurons indexed as #12 (A) and #17 (B). Both spike trains were taken from waking periods. The parabola representing the perfect Gaussian distribution is placed at the bottom of each panel to help readers see the degree of non-Gaussianity. The spike train from #12 is highly non-Gaussian, while that from #17 is weakly non-Gaussian.

The method employed has previously been used and described in Refs [10]–[13]. However, we outline the method here to make the present paper self-contained. We consider a series of ISI's denoted by 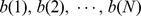 in a given period of observation time (Fig. 1(A)), which we cumulate as 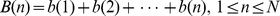, (Fig. 1(B)). For neural firing at a constant rate, 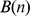 grows linearly, while for firing with some degree of adaptation, 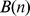 curves up-/downward. Our immediate goal is to determine, at each scale 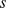, stochastic fluctuations of 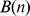 from its smoothly changing tendency, or “trend”. To do this, we first divide the whole observation time into half-overlapping 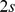-sized segments: 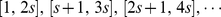, and then determine a polynomial fit to data points within each segment. The fitting error or residual, 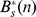, represents the stochastic fluctuation at scale 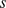 that we sought. This procedure is often referred to as detrending [14]. The stochastic fluctuations cumulated over a time scale of 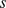 are calculated as 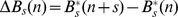. A measuring device which fails to follow too rapid fluctuations might intuitively be thought to display values of 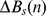 at every 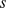 events. A larger time scale corresponds to a lower temporal resolution. Sufficient detrending erases biases in fluctuations, 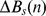, so that it is symmetrically distributed around zero (Fig. 1(C)(D)). In the present study, we used a cubic-polynomial fitting because a higher order detrending did not significantly change the results. Figs. 1(C)(D) display the normalized stochastic variable, 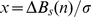, where 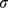 denotes a standard deviation 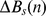. The normalized variables are displayed at two different coarse graining scales, 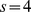 and 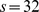. The probability distribution functions (PDF) of the normalized quantity at eight different coarse graining scales, are shown in Fig. 2(A). A sharp peak and a heavy tail of the PDF which represent the non-Gaussianity, are preserved up to the largest scale for this particular neural spike train. The non-Gaussianity surviving at large scales, signals the breakdown of the Central Limit Theorem (CLT). The larger the scale is, the more consecutive ISI's we concatenate to measure the fluctuation sum, 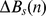. Concatenating statistically independent ISI's, results in 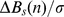 approaching a Gaussian distribution for increasing 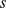. The failure to converge to the Gaussian distribution, as e.g. illustrated in Fig. 2(A), evidences the presence of statistical correlations between different ISI's. Some spike trains showed non-Gaussianity more rapidly diminishing with scale (Fig. 2(B)). However, the majority of spike trains we analyzed showed a very slow convergence to Gaussianity. A complete analysis is provided in the following sections.

The degree of non-Gaussianity can be quantified based on what is known as Castaing's equation, originally developed to characterize the multiscale nature of hydrodynamic turbulence [8]. In this method, the non-Gaussian PDF is represented as a log-normally weighted superposition of Gaussian distributions with different widths, parameterized by non-Gaussianity parameter 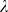:
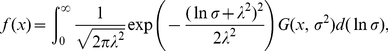
(1)where 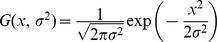. Given that the fitting parameter, 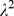, vanishes when the PDF approaches the Gaussian, we confirm that 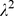 serves as a measure of non-Gaussianity. Interestingly, the multiscale analysis of non-Gaussianity has been shown to work well in characterizing not only turbulence, but also fluctuations in foreign exchange rates [15], stock indexes [12], and human heartbeat intervals [10], [11]. We used the moment based estimator of 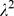 developed in [13], bracketing the moment parameter with 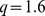 and 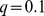.

### Presence of multiple scales in neuronal firing

In the present study, we apply multiscale analysis to ISI's recorded with five multiunit electrodes (tetrodes), each of which has four channels. Five rats that were free from anesthesia were used for our recording. Among five tetrodes, four were inserted into the motor/sensory cortex and one was inserted into the hippocampus, and the spontaneous neural activity was recorded. Using a spike-sorting technique [16]–[18], we separated signals from each of the tetrodes into spike trains of individual neurons. The resultant spike trains were divided into segments “asleep” and “awake”, in accordance with videotaped images of the rats [6]. Multiscale analysis generally requires long series of data. Among neurons of which the spike trains were identified by the procedure, there were 266 neurons which provided over a thousand firing events both in sleeping and waking periods separately.

As illustrated in Fig. 2(A), ISI fluctuations of a number of neurons for the waking animal display strong non-Gaussianity persisting well up to 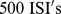. This corresponds to about four minutes for the neuron (#12) whose average firing rate is 2.3Hz. The non-Gaussianity of some other neurons, such as the one shown in Fig. 2(B) (#17), decays more rapidly with a scale. In order to characterize the variety of the size and scale-dependence of the non-Gaussianity, we quantified 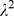 versus 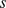 for all the neurons.

**Figure 3:**
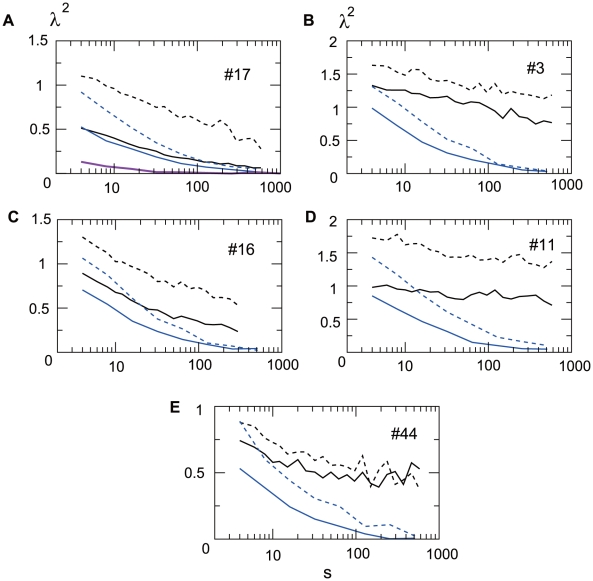
Scale dependence of the non-Gaussianity evaluated. The 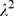 values that quantify the non-Gaussianity are plotted against a non-dimensional scale (number of ISI's) ranging from s = 4 to s = 582. The solid and dashed black lines represent 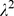 values calculated from spike trains during sleeping and waking, respectively. The blue lines represent 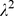 values calculated from spike trains of which the ISI's have been randomly shuffled. Five neurons, #17 (A), #3 (B), #16 (C), #11 (D) and #44 (E), were taken as examples. The non-Gaussianity calculated from a Poissonian spike train is shown as a reference in purple in (A). The non-dimensional maximum scale s = 1000 corresponds with the time scale through individual mean firing rates of neurons. The corresponding time scales are 120s (sleeping) and 130s (waking) for #17, 420s (sleeping) and 630s (waking) for #3, 450s (sleeping) and 590s (waking) for #16, 630s (sleeping) and 430s (waking) for #11, 500s (sleeping) and 590s (waking) for #44.

The panels in Fig. 3 show 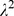 plots of five different neurons indexed as #17, #3, #16, #11, and #4. One of them (#11) is a hippocampal neuron and the others are cortical neurons. The values of 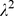 during sleeping and waking are drawn with solid and dashed black lines. In all cases, the 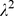 values calculated from the original spike train (black) are higher than those calculated from the randomly shuffled spike train (blue). Also, we note that the 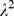 values calculated from the Poissonian spike train (overlaid on the first panel of Fig. 3 in purple) are very small, as expected. The 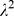 values for the Poissonian spike train serve as a reference “baseline” 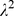 of a memoryless Poisson process. The non-Gaussianity observed, persists for scales over 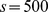, corresponding to several minutes, which is much longer than a typical time scale of the single-neuron dynamics. Interestingly, in the vast majority of cases the non-Gaussianity is consistently stronger during waking than sleeping.

### Non-Gaussianity comes from two sources

**Figure 4:**
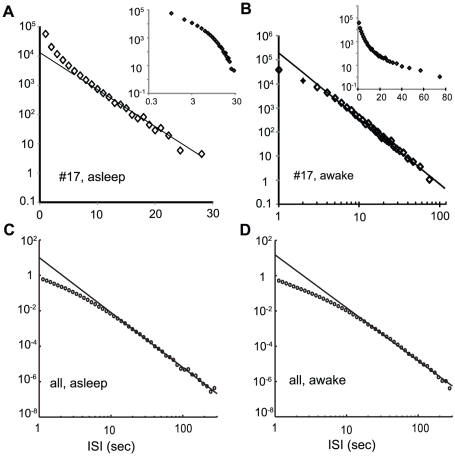
Tails of the ISI distributions. Tails of the ISI distribution of neuron #17 during sleep (A) and waking (B) are plotted either in log-linear or log-log coordinates. The insets illustrate that the alternative plots (log-log in sleeping, log-linear in waking) result in a poorer linear fit in the range where the original plots exhibited a linear tendency. (C)(D) The histograms of ISI's taken during the sleeping (waking) period of all the analyzable spike trains are averaged and plotted in log-log coordinates.

Let us now consider the possible sources of the non-Gaussianity observed. The largely reduced 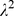 values in the shuffled ISI's suggest the contribution of autocorrelation of the ISI fluctuations to the non-Gaussianity observed. The non-zero autocorrelation implies that each neuron's state eludes the total reset at each spike. The upcoming spike times thus depend on the spiking history. In mathematical terminology, such spike trains are said not to be a renewal process. The autocorrelation of a spike train is, however, not the sole source of the non-Gaussianity. The inherent dynamics of the ISI's is another source of the distribution shape. In fact, even a Poisson spike train of which the autocorrelation is zero has marginal non-Gaussianity, as can be seen in Fig. 3(A) (purple curve). If the ISI distribution of a spike train has a power-law tail, larger non-Gaussianity is expected even without the autocorrelation. This is in stark contrast to the exponential tail of the Poissonian spike train as reported for certain types of neurons [19], [20]. Consistent with the above observation, the 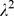 values are large (see Fig. 3(A)) for neuron #17 (waking), of which the ISI distribution has a power-law tail ranging over nearly two decades (Fig. 4(B)). On the other hand, the tail of the ISI distribution of the same neuron during sleep appears exponential (Fig. 4(A)). The exponential decay, as in the Poissonian spike train, is consistent with the observed small 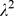 values, see Fig. 3(A). When the ISI histograms during waking, Fig. 4(C), (sleeping, Fig. 4(D)) for all the neurons are averaged, the curve in the log-log coordinate looks linear over an interval that is a little longer than a decade. In summary, the slow decay of the 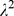 versus 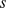 plot is mainly due to the autocorrelation of a spike train, while the large 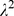 values themselves are also due to the intrinsic distribution shape of the ISI process.

### Autocorrelation of local energy

In order to investigate the direct link between autocorrelation and the decay rate of 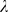, we quantify the autocorrelation of the ISI fluctuation. In our multiscale analysis, we segmented the entire observation time according to scale. For a given scale, we define the local energy of ISI fluctuations [21] as
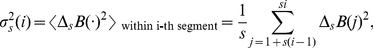
(2)and consider the correlation of the logarithm of this quantity between different segments:
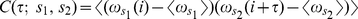
where 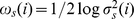, which we refer to as the magnitude correlation [21]. The magnitude correlation is zero if the spike train modulus amplitude (and energy) is not autocorrelated.

**Figure 5:**
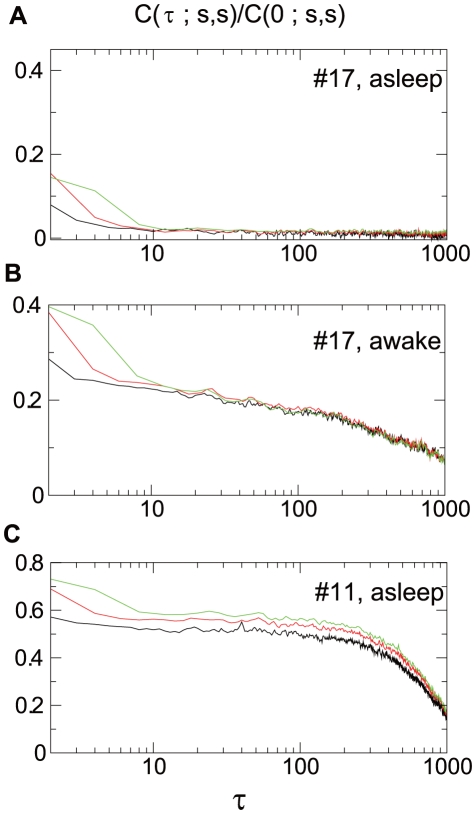
Magnitude correlation functions with 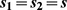. Normalized magnitude correlation, 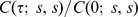, calculated at 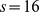 (black), 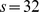 (red) and 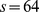 (green) are plotted against non-dimensional distance, 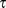. The correlation was calculated for neuron #17 during sleep (A) and waking (B), and neuron #11 during sleeping (C).

The magnitude correlation for spike trains, #17(asleep), #17 (awake) and #11 (asleep) are plotted against 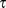 in Fig. 5, whereas 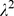 plots for these spike trains are shown in Fig. 3(A),(D). Fig. 5(A) shows that the spike train #17 (asleep) has almost no autocorrelation, which is consistent with the exceptionally small difference of the 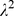 values between the original and shuffled cases in Fig. 3(A)(solid line). The magnitude correlation in a waking state of neuron #17 and a sleeping state of neuron #11 is large, in accordance with the large 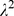 values observed as the dashed line in Fig. 3(A) and the solid line in Fig. 3(D). A remarkable property of near independence of scale of the magnitude correlation is evident in these plots. Such scale-invariance of correlations is characteristic of multiplicative cascade processes and suggests the existence of a scale independent mechanism which is a generating feature for multiplicative cascades [21], examples of which include hydrodynamic turbulence [7] or the financial stock market [12].

### Non-Gaussianity in a population of neurons

Although we performed simultaneous recording of activity from many neurons using a multiunit electrode, our analysis has so far dealt with spike trains from different neurons separately. Here we analyze the entire ensemble of simultaneously recorded multiple spike trains. Neurons detected by one tetrode are up to a hundred micrometers apart [22]. The spiking neurons which we are analyzing are therefore expected to be synaptically connected within such a distance [23].

**Figure 6:**
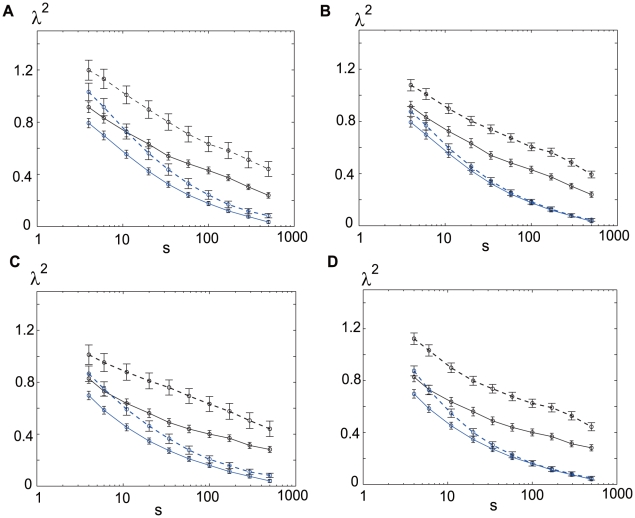
The 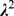 values for a single tetrode and a single neuron are comparable. (A) The 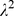 value of a single-tetrode spike train (defined precisely in the text) is calculated for each tetrode. The mean of 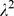 values of 23 tetrodes is plotted in black with error bars indicating the standard errors. The dashed (solid) lines indicate the 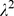 values during waking (sleeping) periods, while the blue lines indicate the 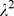 values calculated from randomly shuffled ISIs, in all the panels. (B) The 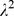 values of a single-neuron spike train are calculated for each neuron and the mean of the 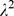 values of 266 neurons is plotted. Panels (C) and (D) are the same as (A) and (B), respectively, except that the moment value, 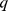, of the 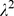 estimator was set to 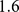 in (A)(B) and to 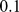 in (C)(D). *p* value calculated by a paired t-test (awake vs asleep) for each case and scale is very small, showing the highly significant wake/sleep difference: (A) 9.9e-05, 5.7e-05, 1.9e-04, 1.0e-03, 4.0e-03, 1.7e-02, 9.8e-03, 1.9e-02, 4.4e-02, 7.2e-02, (B)3.2e-07, 6.4e-08, 8.2e-10, 1.1e-09, 1.6e-13, 8.2e-13, 2.3e-13, 1.4e-14, 3.9e-13, 3.0e-10, (C)2.4e-08, 8.1e-09, 7.0e-09, 4.5e-08, 2.0e-07, 8.3e-06, 3.7e-05, 4.5e-04, 8.5e-03, 3.1e-02, (D)1.4e-25, 4.6e-22, 5.5e-25, 7.1e-21, 8.6e-22, 3.7e-20, 6.8e-19, 1.6e-18, 2.4e-16, 9.9e-11.

Let us consider a “single-tetrode spike train” containing superposed spikes from all the neurons detected by a given tetrode. ISI's for such a single-tetrode spike train can be either inter-spike intervals of successive spikes of the same neuron or the inter-spike intervals between successive spikes of different neurons detected by the same tetrode. We calculate the 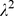 value for each tetrode and average this for all the tetrodes. The results of this procedure are shown in Fig. 6(A).

If the neurons detected by a tetrode fire independently, we expect that the single-tetrode spike train has very low non-Gaussianity (as in the solid line in Fig. 3(A)) because the autocorrelation contained in each single-neuron spike train should be destroyed by the mixing. However, we find in Fig. 6(A) that the 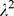 value is comparable to that for the single-neuron spike train, shown for reference in Fig. 6(B), suggesting the presence of the coordinated activity within the population. The 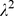 values for this reference Fig. 6(B) are calculated from all the single-neuron spike trains, and they are subsequently averaged over all neurons. The idea of coordinated population activity is further supported by the observation that 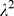 for the single-tetrode spike train becomes largely reduced, if we randomly shuffle the tetrode ISIs (the blue curves in Fig. 6(A)).

We also tested whether our claim of 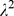 values larger in waking than in sleeping is valid irrespective of the technical detail of 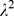 estimation. We repeated the calculations to obtain 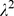 with different values of the moment of the estimator, bracketing the useable interval of 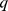 values with 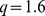 in Fig. 3 and Fig. 6(A)(B) and 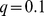 in Fig. 6(C)(D).

**Figure 7:**
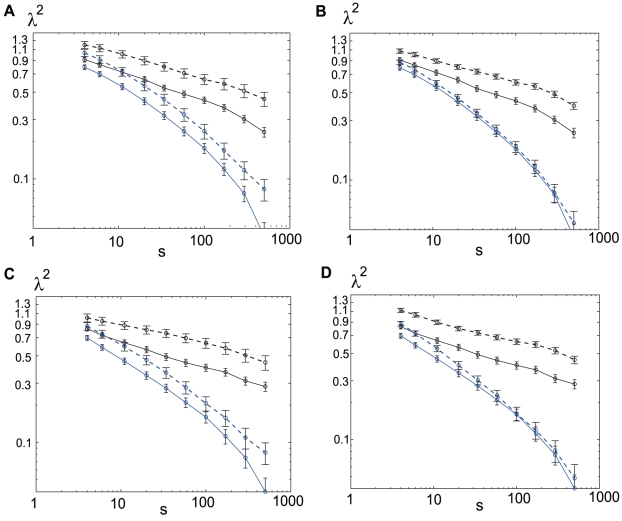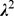 versus 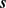 in log-log coordinates. The scale dependence of 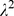 shown in Fig. 6 is replotted in the log-log coordinates.

Throughout this work we argue that the 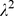 dependence on scale is nearly logarithmic, resulting in a near-linear functional dependence in log-linear coordinates. Such a logarithmic dependence corresponds with classical multiplicative cascades. Yet, in a number of cases the dependence observed could be regarded as a power function of the scale instead of the logarithmic dependence. Indeed, the possibility of the so-called modified multiplicative cascades has been considered both in the fields of fully developed turbulence and in financial analysis [24]. Experimental observations in both these classic examples of cascade phenomena show a departure of 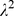 from an ideal logarithmic decay (e.g. Fig. 3a in Ref. [9] and Fig 2b in Ref. [12]). In order visually to verify this in our case, and to permit the possibility of a modified cascade in the spirit of the above phenomena, we also provide plots of the 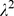 values in the log-log coordinates in Fig. 7. Some of the plots appear better to be described as the power law for more than two decades. This suggests that neural dynamics may require a more complex description, such as in terms of modified hierarchical structures, similar to that found in the turbulence in a jet [9].

## Discussion

In the present study, we have applied multiscale analysis to the ISI fluctuations of spike trains of the cortical and hippocampal neurons of rats in the alternating states of waking and sleeping. As a result, we have found evidence for persistent non-Gaussianity up to a time scale of several minutes.

Different types of multiscale analysis were previously applied to spike trains recorded from the subcortical areas (hippocampal-amygdala complex) of human epileptic patients [25]. Temporal as well as spatial power-law correlations were observed and their significance was discussed. Our experimental set-up with the special chamber enabled us to record cortical activity from healthy subjects for up to eight hours in a row. Such long-term stable recordings provided us with a large enough number of spike events in the cortex, where a generally low firing rate disables the acquisition of sufficiently numerous spikes. Long intervals of high-quality data are essential for multiscale analysis, because it relies on coarse graining to extract statistical properties across a range of time scales. The use of animals, which enables long-term stable recordings of spikes, is therefore a good starting point to open up our understanding of the universal information processing principles in the brain. In waking rats, the degree of non-Gaussianity of spike trains from a single-neuron or a population of adjacent neurons, is well described with the log-linear function of a scale. Interestingly, such a logarithmic decay is a well-known characteristic of fully developed hydrodynamical turbulence or more generally a mathematically defined multiplicative cascade process.

The strength of the non-Gaussianity found in the present study is larger than that observed in any other phenomena previously analyzed using an analogical methodology [10]–[12]. The strong non-Gaussianity may reflect a unique feature of information transmission across the brain, resilient to filtering out despite the filtering characteristics of each neuron.

### Single-neuron mechanism versus network mechanism

As to the possible origin of the multiscale nature of the phenomena observed, we know of several single-cell factors such as intracellular Ca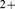 concentration, which may be able to escape resetting after neural firing and hold memory for a longer time scale than the membrane time constant (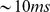). However, the time scale for the Ca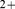 oscillation, reaches only up to tens of seconds [26]–[28], which is not long enough to explain the time scale of several minutes observed here. There still is a possibility of a yet unidentified single-cell factor holding memory for a longer time. However, our analysis of the ISI's of a group of neurons instead of those of a single neuron suggests that the multiple time scales reside not only in a single neuron but also in the network. In fact, the non-Gaussianity level of ISI's of a group of neurons was almost identical to that calculated from the ISI of a single neuron. If the neurons in a group fired independently, implying no network effects being present, non-Gaussianity should become substantially diminished due to mixing. Our observation of the non-Gaussian effects in a population of neurons implies non-trivial network effects that hold at multiple time scales. The lack of good evidence for a single-neuron mechanism, combined with the fact that the non-Gaussianity remains after population mixing, supports the hypothesis that the non-Gaussianity found here is a reflection of spatial correlation or network effects.

Paired patch clamp recordings from the rat cortex have indicated that the connection probability between neurons is high within the distance of 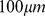 [23]. On the other hand, neurons recorded with a single tetrode are within a hundred 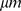 apart [22]. These observations suggest that neurons detected by our single tetrode should be tightly coupled, resulting in both spatial and temporal correlation of neural activity. This observation is consistent with a previous study in the subcortical area [25], which demonstrated an intimate link between temporal correlation and spatial correlation.

### Multiplicative cascade process

The original ancestor of the multiplicative cascade is the binomial cascade introduced by Mandelbrot [19]. The process iteratively redistributes unit mass in proportion (probability) 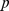 and 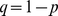 over dyadically, hierarchically subdivided intervals. This procedure produces a multiplicative binomial measure where each sub-interval in the construction has a mass density equal to the product of a sequence of 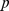
's and 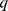
's. Several recent generalizations of the multiplicative cascading process do not require discrete generation steps and have been introduced by a number of researchers [29], also in the context of so-called multifractal random walks [30]. The unifying characteristics of multiplicative cascades are scale invariance of statistical moments, multifractal spectrum of singularities and, of importance to this work, non-Gaussian tails which converge to Gaussianity at a logarithmic rate. A further important characteristic of cascades is that the two-point correlation function is scale-invariant, reflecting the preserved “construction rule” of the cascading process.

History dependent multiple time scale dynamics appear repeatedly in experimental systems at several levels of organization. It is, however, not clear how the multiple time scale dynamics at one level gives rise to multiple scale dynamics at another level. Reference to the multiplicative cascade paradigm may be helpful in elucidating this. Our finding that a neural spike train from the unanesthetized brain is well described by the multiplicative cascade process may suggest that there is a mechanism in the network of neurons in which an observed neuron is embedded, and the spike times of each neuron are determined as a result of hierarchical signal transmission from the largest network structure down to the network elements. In this interpretation, the hierarchical structure is the source of autocorrelation or cross-correlation of spike trains, which we observe directly (Fig. 5) or indirectly (Fig. 3, Fig. 6). Furthermore, a remarkable property of near independence of the magnitude correlation of scale has been established in an average sense. Such scale-invariance of correlations is characteristic of multiplicative cascade processes. It suggests the existence of a scale independent mechanism of correlations, thus a scale independent formation of network memory processes.

### Scale-free activity in neural culture

Segev et al. [2] studied the activity of neurons cultured on MEA and analyzed the positive half of the ISI increment distribution, which was very close, but not identical to the top curves of Fig. 2(B). In fact, 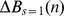 represented the difference between the 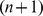 th ISI and the local mean of the ISI, while the ISI increment was the difference between 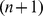 th and 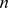 th ISI's. They argued that the tail of the PDF could be fitted with the so-called stable distribution. Our study generalizes and supersedes [2] both in methodology: we considered all the available scales, instead of only 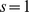, and in neural preparation: we considered the intact brain of an unanesthetized animal instead of culture neurons.

In Ref. [2], the neurons fired in high synchrony across most of the neurons on the preparation, and many of them fired in bursts. For this reason, their firing pattern looked very different from that observed in our in vivo recordings. The histogram of the ISI increment reported in [2] displayed tails extending to long time scales and being cut off at around 100 sec. The cut-off was considerably larger than the neurons' membrane constant.

Segev et al. [2] argued that dynamical synapses, dynamical thresholds of neural firing and inhomogeneous neural resistance were the sources of the long time scales observed. This they supported by reproducing an ISI increment distribution similar to that observed with a network model [31]. It would be fruitful to apply our multiscale method to such neural data from the culture preparation for a closer comparison between their study and ours. It may also be insightful to attempt to construct network models capable of reproducing the type of multiple time scale behavior we observe.

The so-called neural avalanche [3], [32] has recently become one of the most thoroughly studied phenomena involving the scale-free nature of neural activity. Originally, it was found in neurons cultured on MEA [3]–[4], and recently it has also been observed in the superficial layer of the brain of anesthetized rats [5]. The scale-free nature in the spatial dimension is particularly clear-cut, although it is also reported in the temporal dimension. In our case, the direct evidence of the multiscale nature is found in the temporal dimension. However, as we have discussed above, circumstantial evidence supports the multiscale nature even in the spatial dimension. Indeed, both phenomena, that of avalanche processes and cascading processes, unfold in the space-time domain.

### Neural hierarchy

The brain is essentially a multiscale system. The biochemical reactions within the neurons in the brain, on which the entire activity of the brain is based, take place within an order of microseconds to seconds. Most cellular activity takes place at an order of milliseconds, and most cognitive tasks take place within an order of seconds. For instance, phosphorylation of a particular protein is considered to be the most elementary step in forming memory in the brain and it takes place in less than a second of biochemical reactions. The phosphorylated state of the protein lasts for tens of minutes. Our short-term working memory lasts for a similar range of time. However, important memories can be held much more stably. Some memories last a lifetime, and here new transcriptions of certain genes are considered relevant. Such a multiscale nature of brain activity appears to be essential in understanding the brain information processing [33]. The presence of multiple time scales was previously proposed to develop at a more microscopic level as a result of interactions between ion channels and a single neuron [34]. There the hierarchical structure is again considered important.

The present study has found that the non-Gaussianity observed in the living brain matches well to the non-Gaussianity of the multiplicative cascade process. It is therefore tempting to consider further that the hierarchical structure in the brain information processing is also described well by the hierarchical structure that defines the multiplicative cascade process. Such a hypothesis may sound too radical. However, it may provide us with a novel and useful way of looking at the brain, which is too complex to understand from a conventional point of view. Elucidating such a new framework to understand the brain is an important future goal for us. There are studies using a network model simulating the cortex of the brain [35], [36]. Applying the analysis method used here to the activity data from such model networks would be an interesting future direction.

While it is a general result that stochastic multiplicative processes are expected to lead to non-Gaussian behavior, such a multiplicative hierarchy as modeled by cascade processes does not permit the formation of hierarchy loops. Hierarchy is usually imagined as the units organized into a tree-like structure either in space or simply as a connected set of directed links between eventually interacting units. This picture usually involves well defined levels in the hierarchy and no loops within and among the units at the various levels. It would be useful to see to what degree the results support this picture and where a departure from this simplified hierarchical structure seems to be necessary.

A multiscale, hierarchical description, as formalized by the multiplicative cascade paradigm, is of the tree type. This implies correlations and information or causality flow only across the branches of the tree. The dynamical organization of multiscale processes, in particular memory processes in the neural brain networks possibly, or perhaps even likely involves feedback mechanisms across various temporal scales. Such considerations are, however, outside the scope of the current manuscript. Strictly speaking, we believe that such considerations are at present outside the scope of the currently available analysis methodologies. A promising direction may exist in generalizations to the multiscale transfer entropy formulation [37]. Both multivariate and univariate/cross-scale extensions of this formalism would definitely be worth applying to the problem we address.

### Possible influence of spike-sorting errors

Although the spike-sorting algorithm employed here is considered to be of a high quality [18], no spike-sorting algorithm is perfect. Here we discuss the possible influence of spike-sorting errors.

Representative spike-sorting errors are over-division and under-division. Over-division occurs when a single-neuron spike train is mistakenly divided into two or more different spike trains. In the case of under-division, spike trains from different neurons are mistakenly combined into a single-neuron spike train. The spike train of the whole tetrode that we analyzed to obtain Fig. 6(A)(C) represents the maximally under-divided spike train. The similarity between Fig. 6(A)(C) and (B)(D) demonstrates that even the maximal under-division has little influence on our argument in the present study. We next consider a possible influence of over-division. For this purpose, we artificially divide a spike train into two child spike trains in different ways and study how the 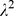 values are changed by the over-division. After calculating 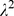 values before and after the artificial over-division, we find that changes in 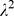 values due to over-division are generally small (supporting figure, Fig. S1). Even when a change in 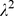 values is significant, we see the general tendency that when the 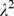 value of one child spike train is larger than that of the original spike train, the value of the other child spike train becomes smaller. Because of this general tendency, which we explain mathematically below, the over-division of a spike train does not create a systematic shift in the 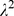 values of the population. Whenever 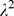 of one component increases, that of the other always decreases. Mathematical proof of this is obtained if we notice that the probability distribution of the ISI fluctuations of the original spike train, 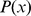, is approximately written as the mean of the corresponding probability distributions for the two child spike trains as 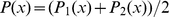. This results in the following relation of the mean values of 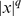: 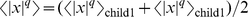. With the 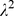 estimator [13], 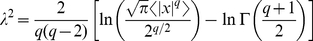, we can conclude that the 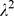 values of the original spike train come in between those of the two child spike trains: 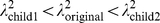.

### Conclusion

Various successful analysis methods developed in physics and applied to biological systems are still mostly single-scale methods. Traditionally, when two or more different time scales exist in a system, the usual practice is to find the separation of time scale and understand the system's behavior at one time scale at a time. This approach works when one time scale is much larger than the other. However, when there is continuum of different time scales, multiscale analysis proves its worth. The present study employs multiscale analysis to study brain activity and reveals the multiscale nature of the spike activity. Our methodology may prove to be a promising new route to understanding the nature of brain information processing.

## Materials and Methods

### Electrophysiological recordings

#### Ethics Statement

All experiments were carried out in accordance with the Animal Experiment Plan approved by the Animal Experiment Committee of RIKEN.

Multiunit recordings were obtained from the somatosensory, motor cortex and hippocampus of adult Long-Evans rats (N = 5; 150–250g, male; Japan SLC, Inc., Hamamatsu, Japan), which were waking and sleeping well in a head-restraint condition for an average period of 7h 40min. A tetrode was inserted into the left somatosensory and motor cortices, and hippocampus up to 1.25 mm in depth. The signals of all the channels were amplified at a 2,000-fold gain, filtered between 0.5 Hz and 10 kHz with a multichannel amplifier (MEG-6116; Nihon Kohden, Tokyo, Japan), and sampled at 20 kHz with a hard-disc recorder (DataMax II; R.C. Electronics Inc., Santa Barbara, CA, USA).

### Spike-sorting

Multiunit recording data were processed to isolate spike events by the semi-automatic spike-sorting method [16]–[18], which is based on the wavelet transform and robust variational Bayesian (RVB) clustering [18], [38], [39]. First, a Mexican-hat type wavelet band-pass filter around 2kHz was applied to raw recording data and spikes were detected by thresholding of filtered data (the threshold set as -6 SD; spike-detection interval 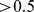
msec). After that, the 18 bimodal wavelet coefficients of the spike waveforms were selected and the coefficients were furthermore reduced to 12 dimensional features using principal component analysis. The extracted features were classified by RVB, and then the classified clusters were combined/discarded manually using Klusters [40].

## Supporting Information

Figure S1 Comparison of the λ^2^ values before and after the artificial over-division. To examine the influence of over-division of a spike train, we artificially divided a spike train the size of which is greater than 4000sec into two “child” spike trains in different ways and studied how the λ^2^ values before (the dashed lines) and after (the pair of solid lines with markers) were different. The three colors correspond to three different neurons. (A)(B) We first determined where to cut the original spike train by drawing uniform random numbers less than d = 500sec, and then divided the original spike train into two child spike trains. The pair of the solid lines represent λ^2^ values of the two child spike trains, while the dashed lines represent the λ^2^ values of the original spike train. Two different random numbers produced (A) and (B). (D)(D) Similar plots for d = 1000sec. (E)(F) Similar plots for d = 2000sec. One can notice that changes in the λ^2^ values due to over-division are in general small. Even when a change in the {lower case lambda}^2^ values is significant, we see the general tendency that when the λ^2^ values of one child spike train are larger than those of the original spike train, the values of the other child spike train are smaller. Because of this general tendency, which is proved mathematically in the text, the over-division of a spike train never creates a systematic shift of the {lower case lambda}^2^ values as a whole. Whenever one has an upward shift, the other always has a downward shift.
(1.01 MB EPS)
